# Plans to accelerate innovation in health systems are less than IDEAL

**DOI:** 10.1136/bmjqs-2015-004605

**Published:** 2015-12-23

**Authors:** Paul M Wilson, Ruth Boaden, Gillian Harvey

**Affiliations:** 1 Manchester Business School, University of Manchester, Manchester, UK; 2 School of Nursing, University of Adelaide, Adelaide, Australia

**Keywords:** Evaluation methodology, Health services research, Evidence-based medicine

## The drive for innovation

Across health systems there is a drive to roll out innovative models of care that will deliver better value for money and improve the quality of care. Innovation in service delivery has been defined as ‘a novel set of behaviours, routines, and ways of working that are discontinuous from previous practice, directed at improving health outcomes, administrative efficiency, cost effectiveness or users’ experience and that are implemented by planned and coordinated actions’.^1^ Undertaking this type of innovation at scale is increasingly viewed as crucial to the long-term sustainability of health systems.

In the USA, the Affordable Care Act has provided a legislative framework that promotes innovation in service delivery. Accountable care organisations (ACOs) have emerged from this as innovative payment and delivery models that aim to improve the coordination and quality of care, enhance population health while containing the growth in healthcare costs.^2^ In England,^3^ ^4^ the Health and Social Care Act has made innovation in the provision of health services a statutory duty and a major initiative to drive innovation at scale is underway.^5^ Fifty ‘Vanguard’ sites are now starting to act as test beds for multicomponent innovations in service delivery (see table 1), supported by a £200 million transformation fund from National Health Service England.

**Table 1:** New models of care: the NHS Vanguards^5^

Integrated primary and acute care systems	Eight test bed sites joining up general practice, hospital, community and mental health services
Enhanced health in care homes	Six test bed sites offering older people better, joined up health, care and rehabilitation services
Multispecialty community providers	Fourteen test bed sites focused on moving specialist outpatient and ambulatory care out of hospitals into the community
Urgent and emergency care	Eight test bed sites developing new approaches to simplify and improve the coordination of services and reduce pressure on emergency departments
Acute care collaborations	Test bed sites (yet to be announced) will link together local hospitals to improve their clinical and financial viability

NHS, National Health Service.

## The danger of innovation without evaluation

The sheer scale of this initiative serves as a timely reminder that innovation without adequate evaluation can lead to misattribution of effects and worse, the wider adoption of technologies, practices and ways of working without proven benefits over existing alternatives. Health systems, and the NHS in particular, can ill afford compromised decision-making in relation to continuation or wider spread on such a scale. Previous large-scale policy initiatives such as the drive to rapidly implement telehealth technologies despite known uncertainties relating to complexity, costs and benefits^6–8^ have led to what might be considered inappropriate allocation of finite resources. While there is some recognition that previous roll out and testing of service innovation has been suboptimal^4^ and that there is a need to establish evidence on ‘what works’,^9^ this on its own will not be sufficient.

Service innovation at scale is inherently complex and context dependent; what may appear to be successful in one setting may not work the same way in a different context.^1^ ^10^ The inter-relationship between the mix of intervention, process, workforce and system changes and the contextual setting within which they are introduced is likely to generate both intended and unintended consequences. Understanding this complexity and its impact on apparent outcomes necessitates more nuanced approaches to evaluation than are currently used. To date, this level of understanding has been lacking in early evaluations of the spending and performance of ACOs,^11^ ^12^ which suggest modest reductions in total Medicare expenditures and differential improvements in the quality of care. Yet the underlying mechanisms within each ACO are complex and evolving and whether or how these will continue to impact on quality and costs in a sustained and positive way or whether they can be reproduced elsewhere is less clear.^13^

## What type of evaluation is needed?

Approaches that are context-sensitive and that address the what, why, how, where and for whom of innovation implementation are necessary if we are to be able to recognise and reproduce ‘success’ at the service level. There is always a tension between rigorous evaluation and ‘good enough’ evidence,^14^ ^15^ and there will always be a trade-off between what evidence is desirable and what is possible. Nevertheless, those implementing complex service innovations should be considering what sort of outcome and process evaluation is necessary from the outset; to do otherwise, would be to reduce value and increase waste.^16^

Prospective pathways for undertaking rigorous outcome and process evaluation of complex and large-scale interventions have been well defined by the UK Medical Research Council (MRC).^17–19^ The aim of these frameworks is to help researchers and funders recognise and adopt appropriate methods to evaluate the systematic and planned introduction of complex interventions. But there are recognised weaknesses within the existing MRC framework, notably the time and resources needed to operationalise it fully.^20^ Others have criticised the focus on the intervention rather than on the dynamic influence of the context into which an intervention is introduced or from which it may emerge.^21^ This lack of acknowledgement that innovation can be unintended as well as intended raises the possibility that other alternative frameworks may be better suited to guiding more nuanced approaches to innovation development and evaluation.

## The IDEAL framework

The IDEAL framework was developed to provide a systematic and incremental pathway to aid the transfer of surgical innovations into practice.^22–25^ The framework presents a five-phase approach to innovation implementation encompassing: idea, development, exploration, assessment and long-term study (see table 2). Although broadly following the MRC framework in its latter phases, IDEAL better recognises the planned and unplanned nature of innovation and the need to adapt methods to the innovation process rather than doing the opposite.

**Table 2:** Phases of the IDEAL framework^22^

Idea	Initial report Innovation may be planned, accidental or forced Focus on explanation and description
Development	Rapid iterative modification of innovation Small experience from one centre Focus on technical details and feasibility
Exploration	Innovation now more stable Replication by others Focus on assessing potential benefits and adverse effects
Assessment	Innovation gaining wide acceptance Considered as possible replacement for current treatment, ways of working Focus on comparative evaluation against current standards or best practice
Long-term monitoring	Long-term surveillance of the innovation Focus on monitoring late and rare problems, changes in use

IDEAL was developed to confront problems with the evaluation of surgical procedures but as the uncontrolled introduction of innovations is not specific to surgery, there is increasing interest in its potential value beyond this field. The Food and Drug Administration has been working with IDEAL to explore how best to advance the infrastructure and methodology for evaluating medical devices.^26^ In England, IDEAL is also being promoted as a tool to help commissioners (payers) to consider requests for funding for novel services and make other resource allocation decisions (about interventions not routinely funded) in a more objective and systematic way.^27^ ^28^ IDEAL therefore has the potential to be adapted and applied to a wide range of situations in which complex innovations are in need of rigorous development and evaluation.

With new models of care some interventions, processes and ways of working are likely to be planned from the outset but others may emerge by accident or occur through the unintended consequences of planned innovation. A focus on explaining early intentions and describing what actually happens over time is therefore key. The idea and development phases of the IDEAL framework recognises this and advocates the iterative development and evaluation of innovations to define and test whether it is likely to succeed in a particular setting and allow for adaptation, refinement and system integration before more widespread and rigorous testing is pursued.^23^

With this initial focus on iterative development, IDEAL also recognises that rapid cycle evaluative approaches that traditionally feature in quality improvement initiatives may be most appropriate in these early phases.^29^ Common to these approaches is the generation of real-time data driven learning and decision-making. This makes them ideally suited to addressing questions such as ‘Should we do this again?’ and ‘Should we pursue this further?’ Approaches such as plan-do-study-act and statistical process control also have the benefit of being widely used as part of efforts to improve the quality and safety of care across health systems. But if iterative evaluation of this type is to be pursued, well documented shortcomings in planning, execution, analysis and reporting of these methods have to be avoided or their effects minimised.^30–32^

And this is where one of the key strengths of IDEAL can best be harnessed; that of transparency through registration and reporting. IDEAL recommends that all surgical innovations are prospectively registered, and that it is unethical not to do so.^22^ In England, infrastructure already exists to facilitate the prospective registration of innovations although the extent to which it is used is unclear, and it may not be intended for service innovations.^33^ For example, at present it is unclear whether a comprehensive register exists of areas of England where ‘integrated care’ in various forms and at different scales is being attempted. Prospective registration will aid transparency by providing a permanent record of how an innovation was intended to be introduced, whether it was adapted and how it was ultimately implemented in a given context.

IDEAL also recommends that the early development and evaluation of any innovation should be adequately reported (regardless of ‘success’ or ‘failure’). Insights and contextual information which could inform wider spread and learning should be systematically captured in a standardised format. There are now a vast number of reporting frameworks available but given the focus on reporting initial innovation development, refinement and implementation, the SQUIRE2 guidelines may be the most appropriate.^34^ Although designed for quality improvement, SQUIRE2 recognises that innovation can be complex, multidimensional and is inherently context dependent. With a focus on explaining issues such as ‘Why did you start?’, ‘What did you do?’, ‘What did you find?’ and ‘What does it mean?’, SQUIRE2 encourages more nuanced reporting of what happened over time and reflection on the meaning of the outcomes and events that were observed.

Beyond the idea and development phases, IDEAL recommends that plans for wider spread should be accompanied by robust and independent comparative evaluation. The small-scale iterative evaluations of the early phases can only ever give tentative indications of benefit and can be prone to misinterpretation and bias; as such they should only ever represent a starting point. Further corroboration, explanation and evaluation should be sought along the IDEAL pathway and prospective comparative quasi-experimental designs that will generate economic, outcome and process data should be the preferred method of evaluation. Here IDEAL recognises and adopts the methods advocated by the MRC for evaluating complex interventions.^17–19^

## Adapting IDEAL

As IDEAL was originally devised for surgical innovations it does have shortcomings when considered in relation to other complex and emergent innovations. One such shortcoming is the failure to advocate the use of clearly articulated theory to explain how and why an innovation would be expected to deliver benefits over existing practices. This may be due in part to the fact that in surgery the theory is usually implicit in the innovation itself. But with increasing complexity (surgical or otherwise) theory becomes an essential lens through which we can predict, identify and describe the key features that will influence the implementation of any innovation.^35^ ^36^ Particularly important is ensuring that there is clarity about what the innovation is together with clarity over the wider system, process and contextual features that will be need to be addressed if its causal mechanism is to function as planned. Encouragingly, use of logic models is being promoted and constructed for each Vanguard site. But this clarity over purpose should also inform the selection of evaluation methods that reflect an innovation's phase of development.^37^ Applying theory in the idea and development phases should help to focus attention on those evaluation strategies that are best equipped to capture innovation adaptation and refinement over time. Doing so will also offer a more efficient and meaningful method to generalise and predict the potential for successful replication in other settings.

Another major shortcoming with IDEAL is consideration of cost. Although IDEAL incorporates cost effectiveness in its latter phases, it is absent from the idea and development phases. Yet innovation (large or small) is not without cost or consequences. There will be opportunity costs as innovation competes with other health activity and services for finite resources. Determining whether the expected gains in health benefits from innovation implementation will exceed those lost as other activities are displaced is therefore crucial.^38^ This tension has been perfectly illustrated by a recent analysis of the impact of making routine hospital services available 6 days a week in England; the costs of the proposed solution reportedly outweigh the health benefits that are likely to be realised.^39^ Early assessment therefore may help to identify and eliminate cost-ineffective innovations. At the very least, innovators should be encouraged to systematically capture and report resource use and costs associated with early stage implementation from a clearly stated perspective—especially important if judgements are to be made on wider spread.

## Conclusions

An adapted IDEAL framework offers a pathway for prospective longitudinal evaluation of the Vanguard test bed sites and similar complex and emergent innovations. Adopting a common analytical framework will encourage iterative development, reporting and evaluation of innovations from the outset and before more widespread and rigorous testing is pursued.
